# Fungi Indirectly Affect Plant Root Architecture by Modulating Soil Volatile Organic Compounds

**DOI:** 10.3389/fmicb.2018.01847

**Published:** 2018-08-13

**Authors:** Denis Schenkel, Jose G. Maciá-Vicente, Alexander Bissell, Richard Splivallo

**Affiliations:** ^1^Institute for Molecular Biosciences, Goethe University Frankfurt, Frankfurt, Germany; ^2^Integrative Fungal Research Cluster, Frankfurt, Germany; ^3^Institute of Ecology, Evolution and Diversity, Goethe University Frankfurt, Frankfurt, Germany

**Keywords:** *Fusarium*, *Arabidopsis*, volatile organic compounds, plant-microbe interactions, soil VOCs

## Abstract

The plant-growth modulating effect of microbial volatile organic compounds (VOCs) has been demonstrated repeatedly. This has most often been performed by exposing plants to VOC released by microbes grown on nutrient rich media. Here, we used soil instead to grow fungi of the *Fusarium* genus and investigate how VOCs emitted by this system influenced the development of *Arabidopsis* plants. The volatile profiles of *Fusarium* strains grown in soil and malt extract were also compared. Our results demonstrate that distinct volatile signatures can be attributed to different *Fusarium* genetic clades but also highlight a major influence of the growth medium on volatile emission. Furthermore, all soil-grown *Fusarium* isolates increased primary root length in *Arabidopsis* by decreasing VOC concentrations in soil. This result represents a major paradigm shift in plant-microbe interactions since growth modulating effects have been attributed so far to the emission and not the consumption of volatile signals.

## Introduction

Volatile organic compounds (VOCs), small molecules with low boiling point and high vapor pressure, include numerous signals involved in plant-microbial interactions (Junker and Tholl, 2013; Schulz-Bohm et al., 2017). To date, a few thousands VOCs have been described in flowering plants (Knudsen et al., 2006) and microbes (Lemfack et al., 2018). These VOCs predominantly include terpenoids, phenylpropanoids/benzenoids, fatty acids, and amino acid derivatives (Dudareva et al., 2013). By contrast to their well-established functions aboveground (Šimpraga et al., 2016), the role of VOCs released belowground has only started to emerge recently.

Rhizosphere microbes might regulate plant growth by emitting VOCs as demonstrated by *Bacillus* bacteria (Ryu et al., 2003; Zhang et al., 2007) that promote plant growth through the release of 2,3-butanediol and acetoin (Ryu et al., 2003). Similar growth promotion in *Arabidopsis* was reported for *Trichoderma viride* fungi with growth regulators putatively identified as isobutyl alcohol, isopentyl alcohol and 3-methylbutanal (Hung et al., 2013). VOCs emitted by the fungal root pathogen *Rhizoctonia solani* have also been shown to increase shoot and root biomass in *Arabidopsis* and at the same time compromising resistance to above-ground herbivory (Cordovez et al., 2017). Microbial VOCs might also inhibit plant growth and affect root development as documented for bacteria (Wenke et al., 2012) and mycorrhizal fungi (Splivallo et al., 2007; Ditengou et al., 2015). These examples illustrate the seemingly wide-spread ability of rhizospheric microbes to regulate plant development through VOCs. Regulation of plant growth by secondary metabolites occurs in a concentration-dependent manner, with promotion typically reported at low concentrations and inhibition at higher concentrations as demonstrated for benzaldehyde derivatives (Choi et al., 2016). Bioactive concentrations mimic hormonal levels as shown for some VOCs (i.e., 13-tetradecadien-1-ol, 3-hydroxy-2-butanone, 2,3-butanediol) that promote plant growth in the ng-μg range for microbes grown on non-soil matrices (Ryu et al., 2003; Zou et al., 2010; Blom et al., 2011; Park et al., 2015). As most media used in previous studies are rich in terms of nutrients, it might be more challenging for organisms growing on soil to produce secondary metabolites. In bulk soil and the rhizosphere, bioactivity will eventually depend on the half-life of specific signaling molecules as well as the ability of the emitter to sustain stable VOCs concentrations over longer periods of time.

In terms of phylogeny, the ability to regulate plant-growth via VOCs seems widespread among diverse microbial species (Sánchez-López et al., 2016) and microbial VOCs might act by modulating the auxin and the cytokinine signaling pathways of plants (Ryu et al., 2003; Zhang et al., 2007; Bitas et al., 2015; Sánchez-López et al., 2016). Volatile signals of *Trichoderma* fungi have been shown to increase the uptake of iron in roots of *Arabidopsis* and tomato, resulting in the priming of jasmonic acid signaling pathway in plant shoots (Martínez-Medina et al., 2017). It has also been suggested that microbes occupying particular niches (i.e., rhizosphere) or having specific lifestyles (ectomycorrhizal, pathogenic or saprophytic) might produce distinct ecologically relevant VOCs (Müller et al., 2013; Schenkel et al., 2015). If confirmed, this hypothesis might corroborate the importance of specific VOCs in plant/microbial interactions.

The influence of microbial VOCs on plant growth has been tested in various static and flow-through systems where plants and microbes are physically separated but exchanged VOCs via a common headspace (Kai et al., 2016). Static systems such as a split Petri dish have been widely used, with plants in one compartment and microbes in a second compartment (Blom et al., 2011; Kai et al., 2016; Lazazzara et al., 2017). One shortcoming of the static systems described previously is that they used artificial (nutrient rich) media to grow microbes. VOCs profiles emitted by microbes grown on nutrient rich media might drastically differ from those emitted under more natural conditions (i.e., with microbes grown on soil), which might in turn affect plant phenotype. Thus growth conditions should be carefully selected when investigating potential ecologically relevant signaling compounds. Another shortcoming of static systems is that they are prone to the artificial accumulation of humidity (Tholl and Röse, 2006; Kai et al., 2016) and CO_2_ (Kai and Piechulla, 2009) that must to be controlled for unbiased results (Piechulla and Schnitzler, 2016). Ultimately, bioactive VOCs must be identified and quantified, and their plant growth regulation ability should be demonstrated with pure compounds in concentrations similar to the ones observed in the assays with plants.

The first aim of our study was to investigate the influence of growth medium (a nutrient rich medium versus soil) on the emission of fungal volatiles. The second aim was to test the outcome of VOC-mediated interactions between plants and soil-grown fungi. For this purpose, we employed *Arabidopsis thaliana* as a model plant and different species belonging to the *Fusarium* genus. This genus comprises globally distributed ascomycete fungi of the Hypocreales order (Karim et al., 2016) that range in lifestyle from plant pathogens and saprophytes (Karim et al., 2016) to symptomless endophytes (Maciá-Vicente et al., 2008; Glynou et al., 2016). Members of the *Fusarium* genus cannot be strictly classified as saprophytes or pathogen since their lifestyle is highly dependent on the colonized host plant and possibly other environmental conditions (Kia et al., 2017; Lofgren et al., 2018). Previous work on *F. oxysporum* grown on nutrient rich media highlighted its ability to alter plant growth via the emission of VOCs (Bitas et al., 2015). In contrast to the latter study, soil was employed here as substrate for fungal growth. Additionally, further species of the same genus were tested to understand whether general patterns of VOC-mediated growth regulation could be inferred from phylogenetic data.

## Materials and Methods

### Identification of Fungal Strains

Twenty-eight fungal strains, including twenty-seven *Fusarium* (Hypocreales) and one unclassified Helotiales strains were used in this study. All strains were isolated as endophytes from the roots of two species of the Brassicaceae family, *Microthlaspi perfoliatum* and *Microthlaspi erraticum* originating from several locations in Europe and Turkey (Glynou et al., 2016) (**Table 1**).

**Table 1:** *Fusarium* and Helotiales strains used in this study.

Strain number	Species	Country	NCBI GenBank ITS accession	NCBI GenBank *tef1-α* accession	Experiment^∗^
P1006	*F. equiseti*	Turkey	KT268325	MH093655	VOC_ME_
P1007	*F. acuminatum*	Turkey	KT268326	MH093656	VOC_ME_
P1013	*F. redolens*	Turkey	KT268332	MH093657	VOC_ME_
P1065	*F. acuminatum*	Turkey	KT268384	MH093658	VOC_ME_ _+SOIL_ / Bioassay
P1076	*F. equiseti*	Turkey	KT268395	MH093659	VOC_ME_
P1078	*F. acuminatum*	Turkey	KT268397	MH093660	VOC_ME_
P1112	*F. acuminatum*	France	KT268430	MH093661	VOC_ME_
P1141	*F. oxysporum*	France	KT268459	MG570086	VOC_ME_ _+SOIL_ / Bioassay
P1143	*F. oxysporum*	France	KT268461	MH093662	VOC_ME_
P1144	*F. oxysporum*	France	KT268462	MH093663	VOC_ME_
P1185	*F. oxysporum*	Croatia	KT268501	MH093664	VOC_ME_ _+SOIL_ / Bioassay
P1286	*F. oxysporum*	Spain	KT268581	MH093665	VOC_ME_ _+SOIL_ / Bioassay
P1304	*F. acuminatum*	Spain	KT268599	MG570084	VOC_ME_
P1388	*F. flocciferum*	Spain	KT268683	MH093666	VOC_ME_
P1431	*F. acuminatum*	Spain	KT268725	MH093667	VOC_ME_ _+SOIL_ / Bioassay
P1473	*F. tricinctum*	Croatia	KT268767	MH093668	VOC_ME_
P1520	*F. tricinctum*	Croatia	KT268725	MH093669	VOC_ME_ _+SOIL_ / Bioassay
P1546	*F. equiseti*	Croatia	KT268840	MH093670	VOC_ME_
P1547	*F. tricinctum*	Croatia	KT268841	MH093671	VOC_ME_
P2134	*F. culmorum*	France	KT269397	MH093672	VOC_ME_
P2186	*F. redolens*	Greece	KT269449	MH093673	VOC_ME_ _+SOIL_ / Bioassay
P2190	*F. flocciferum*	Greece	KT269453	MG570085	VOC_ME_
P2191	*F. torulosum*	Greece	KT269454	MH093674	VOC_ME_
P2194	*F. equiseti*	Greece	KT269457	MH093675	VOC_ME_
P2195	*F. oxysporum*	Greece	KT269458	MH093676	VOC_ME_
P6053	*F. acuminatum*	Spain	KT270246	MH093677	VOC_ME_
P6075	*F. oxysporum*	France	KT270268	MH093678	VOC_ME_
P1176	Unclassified Helotiales	Croatia	KT268493	Not available	Bioassay

*^∗^The experiment column indicates whether a fungal strain was used for VOC profiling on malt extract (VOC_ME_), VOC profiling on soil (VOC_SOIL_), or for testing VOC-mediated interactions with Arabidopsis (Bioassay).*

The strains, characterized earlier (Glynou et al., 2016) by internal transcribed spacer (ITS) sequencing, were further characterized here through sequencing of the transcription elongation factor 1-α gene (*tef1-α*), which provides a better species resolution for *Fusarium* spp. than the ITS (O’Donnell et al., 2015). Using primers ef1/ef2 and PCR conditions reported earlier (Geiser et al., 2004), the partial *tef1-α* gene was amplified from fungal DNA (Glynou et al., 2016). The resulting amplicons were purified and sequenced by GATC Biotech AG (Konstanz, Germany). The quality-filtered sequences were first manually compared against the FUSARIUM-ID database (Geiser et al., 2004) using BLAST (Altschul et al., 1990). Then a maximum-likelihood (ML) phylogenetic tree was built alongside sequences from reference strains. To do this, all the strains’ *tef1-α* sequences were compared using BLAST against the full NCBI GenBank database and against subsets of it containing only sequences from vouchered strains or species type specimens. A maximum of ten BLAST best hit sequences were retrieved from each dataset and aligned with the query sequences applying the G-INS-I algorithm of MAFFT v7.123b (Katoh and Standley, 2013). The alignment was trimmed with Gblocks v0.91b (Castresana, 2000) and then a ML tree was constructed with the program RAxML v8.0.0 (Stamatakis, 2014), using a GTRGAMMA model of nucleotide substitution and rate heterogeneity and 1,000 bootstraps. Another ML tree was likewise built using only the strain sequences, to compare the phylogenetic relationships among strains with their VOC production profiles.

### Experimental Design and Preparation of Fungal Inocula

**FIGURE 1:**
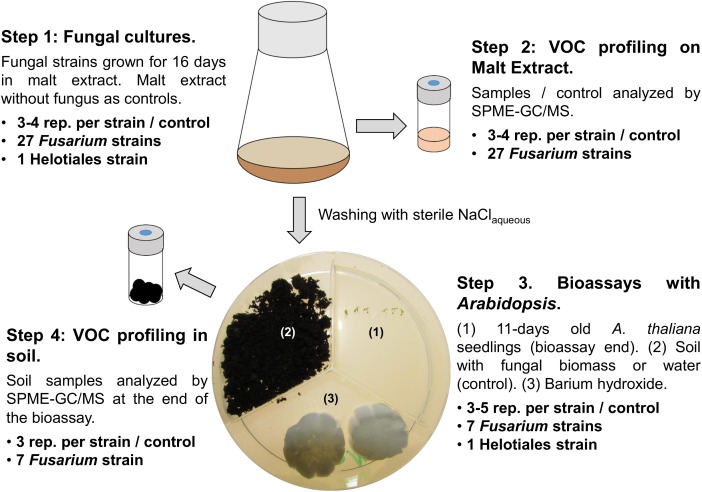
Experimental design. The figure illustrates the four experimental steps as well as the specific fungal strains and numbers of replicates used for each experiment. Step 1: Fungal strains were grown in liquid medium; sterile medium was used as control. Step 2: Fungi were harvested by centrifugation, one part of the mycelium or control medium was analyzed by GC/MS, one part was used to inoculate sterilized soil for the bioassays (step 3). Bioassay setup: six seeds of *A. thaliana* were grown exposed to VOCs released from the soil compartment. Plant morphology was determined at the end of the experiment and soil samples were analyzed by GC/MS (step 4).

Fungal strains, maintained on corn meal agar (CMA, Sigma-Aldrich, St. Louis, MO, United States) at room temperature, were used in a series of experiments aimed at characterizing VOC profiles and VOC mediated interactions of fungi and plants (see **Figure 1** for an overview of the experimental design).

Fungal inocula for VOC profiling and bioassays were prepared by growing each strain for 16 days in 100 ml Erlenmeyer flasks containing 30 ml of 15 gl^-1^ malt extract broth, pH 7.0 (Becton, Dickinson and Company, Heidelberg, Germany) at 24°C (step 1, **Figure 1**). Resulting fungal VOC profiles were characterized by gas chromatography/mass spectroscopy (GC/MS) (step 2, **Figure 1** and “VOC profiling” hereafter) while the remaining fungal biomass was used to test the effect of VOCs emitted by selected *Fusarium* and the Helotiales strains on the model plant *Arabidopsis thaliana* ecotype Col-0 (*Arabidopsis* onward; step 3, **Figure 1**). These assays were also employed to profile the VOCs emitted by *Fusarium* grown in soil (step 4, **Figure 1**). Details about each experimental step is given hereafter.

### VOC Profiling

Volatile organic compound profiling was performed in 20 ml solid-phase micro extraction (SPME) vials for fungal cultures or soil samples. Specifically, samples in malt extract contained either pelleted fungal biomass (1.25 g harvested after centrifugation at 12,000 ×*g* for 10 min) or culture medium without fungal inoculum (1.00 ± 0.05 ml). Alternatively, samples contained 1.50 ± 0.05 g of soil either inoculated with *Fusarium* or without fungus (control), taken directly at the end of the bioassays from the plate harboring soil (refer to “Bioassays with plants and fungi grown in soil”).

Solid-phase micro extraction vials, sealed with a silicon/polytetrafluoroethylene septum, were incubated at 60°C for 20 min prior to VOC sampling. VOCs from the head-space were subsequently extracted for 15 min at 60°C using a Divinylbenzene/Carboxen/Polydimethylsiloxane SPME fiber (DVB/CARB/PDMS) pre-conditioned following the manufacturer’s instructions (Sigma-Aldrich, Munich, Germany). Empty vials were regularly included in between runs to check for carry-over and at the beginning of each run to generate a blank. GC/MS analysis was performed on an Agilent 7890B GC coupled to an Agilent 5977B MSD (Agilent, Waldbronn, Germany). VOCs were desorbed from the SPME fiber at 250°C (GC inlet temperature) in splitless mode and were separated on an HP-5ms Ultra Inert capillary column (30 m × 0.25 mm with 0.25 μm coating) using the following program: 40°C for 5 min, ramp 3°C min^-1^ until 160°C, ramp 50°C min^-1^ until 260°C, hold 260°C for 6 min. Helium was used as a carrier gas (1.2 ml min^-1^). The MS scan range was 50–350 atomic mass units and ionization was performed at 70 eV. An EI 350 XTR was used as MS source. Source temperature was 230°C, MS quad temperature 150°C.

Chromatograms derived from the GC/MS analysis of fungal cultures and soil samples were aligned with the TagFinder 4.1 software (Luedemann et al., 2008) using parameters described earlier (Sherif et al., 2016), with the exception of the threshold which was set to 500. This resulted in a data matrix comprised of TAGs equivalent to single m/z values within a specific time range [(m/z, RT range)]. TAGs (made of one or more VOCs) present in empty SPME vials were subtracted from the data (3× blank removal). The data were normalized by dividing specific TAGs in each sample by the sum of all TAGs in that sample. This procedure is equivalent to normalizing the peak areas of single VOCs to the Total Ion Current (TIC).

Volatile organic compounds in TAGs of interest were either tentatively identified by comparison of their mass spectra to the ones found in the NIST Mass Spectral Search Program 2.0 (National Institute of Standards and Technology, Gaithersburg, MD, United States) or fully identified based on MS spectra, Kovats retention indices (n-alkane) and injection of an authentic standard. Specific VOCs that were fully identified were purchased from Sigma-Aldrich and Merck (Darmstadt, Germany), including (CAS number in parenthesis): furfural (98-01-1), 4-heptanone (123-19-3), 5-methyl-3-heptanone (541-85-5), benzaldehyde (100-52-7), 5-methyl-2-furancarboxaldehyde (620-02-0), 3-octanone (106-68-3), octanal (124-13-0), 2-ethyl-1-hexanol (104-76-7), benzeneacetaldehyde (122-78-1), nonanal (124-19-6), camphor (76-22-2), 1,2-dimethoxy-benzene (91-16-7) and decanal (112-31-2).

### Quantification of VOCs Emitted From Soil

The concentration of seven VOCs (namely benzaldehyde, decanal, 5-methyl-2-furancarboxaldehyde, furfural, 5-methyl-3-heptanone, 2-ethyl-1-hexanol, nonanal) were quantified in soil by standard addition. These seven compounds were selected based on a linear model suggesting that they might be responsible for the root shortening of *Arabidopsis* observed in non-inoculated soil (see for details “*Fusarium* spp. decrease the emission of soil VOCs in the results section). For this purpose, water was first added to dried soil to adjust water content to the one determined at the end of the bioassays with *Arabidopsis*. The resulting sample (1.50 g wet soil = 0.26 g soil + 1.24 g water) was placed into a 20 ml SPME vial, and spiked either with 20 μl pure dichloromethane, or 20 μl dichloromethane with varying concentrations of single VOCs. Quantification was obtained by calculating the x-intercept of the resulting linear calibration curves.

### Bioassays With Plants and Fungi Grown in Soil

Bioassays in tripartite Petri dishes (Greiner Bio One, Frickenhausen, Germany) were used to test the influence of fungi on root development. The partitioning in the Petri dishes ensured that interactions among organisms were strictly mediated by VOCs (see the illustration in **Figure 1**). The setup was based on Kai and Piechulla (2009), reviewed in Piechulla and Schnitzler (2016) and Kai et al. (2016). One compartment of each plate contained sterile soil inoculated with a single fungal strain (or non-inoculated as control). A sterile cotton piece with barium hydroxide (3 ml of a 25.7 g l^-1^ BaOH aqueous solution) was placed in another compartment to mitigate CO_2_ buildup (Piechulla and Schnitzler, 2016), and the third compartment harbored six seeds of *Arabidopsis*.

The plant compartment of the tripartite plates was filled with half strength Murashige and Skoog basal medium with 15 g l^-1^ sucrose (M9274, Sigma) and 1.5% agar (w/v). The *Arabidopsis* seeds were surface-sterilized for 2 min with 70% ethanol (v/v) followed by 20 min in a 1% sodium hypochlorite (NaHClO_3_) solution in water (v/v) and rinsed three times with sterile water. Seeds on Murashige and Skoog medium were stratified for 2 days at 4°C prior to the bioassays. Thereafter, the remaining Petri dish compartments were completed with barium hydroxide and soil/fungal biomass. Specifically, twice autoclaved commercial soil (Vermehrungssubstrat C200, Stender AG, Schermbeck) (4 g) was added to one compartment of the Petri dish.

**FIGURE 2:**
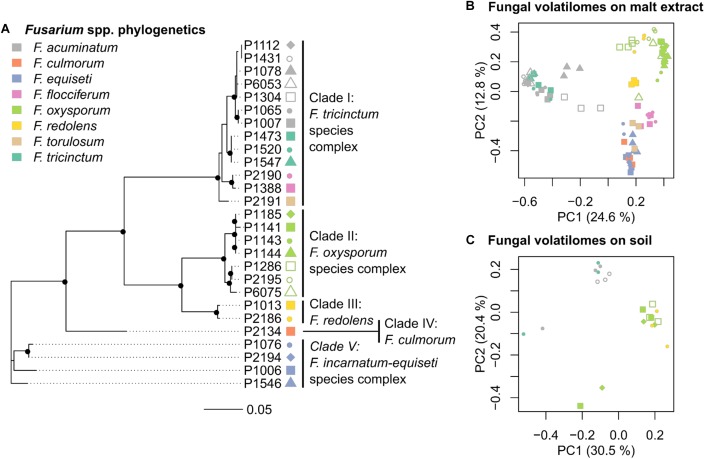
Phylogeny and volatilome of *Fusarium* spp. **(A)** Phylogenetic tree of 27 Fusarium strains based on partial *tef-1α* sequences (**Table 1**). Black dots in the tree nodes indicate bootstrap values ≥70% based on 1,000 replicates. **(B)** Principle Coordinate Analysis (PCoA) based on the VOC profiles of 27 *Fusarium* strains grown in malt extract (*n* = 3–4 replicates per strain) **(C)** PCoA based on the *VOC* profiles of 7 Fusarium strains grown in soil (*n* = 3 replicates per strain). Each point in PCoAs correspond to an independently measured volatile profile, with point color indicating the species (see color key), and different point types within each clade denoting different strains. Both PCoAs highlight that differences in *VOC* profiles among strains correspond by large to genotypic differences.

To test the effect of fungal volatiles on early plant development, mycelium pre-grown for 16 days of one of seven selected *Fusarium* strains was placed on soil in the remaining compartment (**Figure 1**). These seven strains were selected from the three genetic clades I, II, and III (**Figure 2A**) based on the similarities and differences observed on their VOC profiles on malt extract (**Figure 2B**). To evaluate specificity of effects observed, one Helotiales strain was included in the bioassay. Fungal biomass (**Figure 1**, step 1 and **Table 1**) was homogenized for 1 min with a blender (IKA T18 digital Ultra-Turrax IKA-Werke GMBH, Staufen, Germany), washed twice with 0.85% (w/v) NaCl_aq_ to remove traces of malt extract, and pelleted by centrifugation at 12,000 ×*g* for 10 min. A wet fungal biomass of 50.0 ± 0.5 mg was transferred to fresh tubes and resuspended in 1 ml 0.85% (w/v) NaCl_aq_. These were homogenously distributed on the soil surface (**Figure 1**, Step 3). The same volume of 0.85% (w/v) NaCl_aq_ without fungus was used for control Petri dishes.

Plates, sealed with Parafilm, were incubated in an upright position in a growth chamber (Binder KBW 400, Tuttlingen, Germany) for 11 days (23°C, a 16 h photo period, 8,140 lux). Plates were digitalized at the end of the assays with a flat-bed scanner and leaf size and primary root length were measured with the ImageJ (v1.51q) software (Schneider et al., 2012). Secondary root branching was determined under a stereomicroscope (Leica MZ16, Leica Microsystems, Wetzlar, Germany).

### Bioassays With Plants and Synthetic VOCs

Bioassays with mixtures of seven synthetic VOCs were performed similarly to the ones with soil, except that soil was replaced with a piece of sterile cotton harboring mixtures of synthetic VOCs in 20 μl dichloromethane. The mixture of compounds tested in these assays were benzaldehyde, decanal, 5-methyl-2-furancarboxaldehyde, furfural, 5-methyl-3-heptanone, 2-ethyl-1-hexanol, and nonanal. Specific mixtures contained 0.00015, 0.15, 1.5, 10, 20, and 100 μg of each volatile compound in 20 μl dichloromethane. Quantities were selected to reflect the concentrations of single VOCs determined in soil (**Table 2**). These mixtures were added to the Petri dish at the beginning of the bioassay and once again after 6 days. Control Petri dishes were prepared with 2× 20 μl dichloromethane similarly added at the start of the assay and on day six. Plant morphology was evaluated as in the earlier assays after eleven days of exposure to VOCs.

**Table 2:** Quantity of selected VOCs in soil at the end of the bioassays.

VOCs	CAS number	R^2^ linear fit for calibration curve	Concentrations in soil [μg^-1^ soil (wet weight)]
2-Ethyl-1-hexanol	104-76-7	0.95	25.91 ^∗^ 10^-3^
Furfural	98-01-1	0.99	15.64
5-Methyl-2-furancarboxaldehyde	620-02-0	0.98	16.19
Nonanal	124-19-6	0.99	1.01
Decanal	112-31-2	0.85	2.16
Benzaldehyde	100-52-7	1.00	1.11
5-Methyl-3-heptanone	541-85-5	0.99	0.10–0.16

### Statistical Analysis

Statistical tests were conducted at several stages using Past 3.04 (Hammer et al., 2001) or R version 3.4.3 (R Core Team, 2017) and packages within. Nonparametric Kruskal-Wallis tests were performed in Past to identify VOCs (TAGs) which concentrations significantly differed among samples and to assess differences in plant morphology (*Arabidopsis* primary root length, leaf size and root branching) resulting from different treatments. Correlation of VOCs and *Arabidopsis* primary root length was tested via a linear model with Past. Boxplots of primary root length were generated with R, as well as phylogenetic trees (using the package “ape” 5.0 (Paradis et al., 2004)) and Principle Coordinate analyses (PCoAs) (using the package “vegan” 2.4-5 (Oksanen et al., 2017)). For PCoAs all VOC matrices were square root transformed. Details about statistics and number of replicates are given in figure legends.

## Results

### Fungal VOC Profiles Depend Both on Genotype and Culture Media

The first objective was to genetically characterize fungal isolates to relate genotypic diversity to variability in VOC profiles. Partial sequencing of the *tef1-α* gene indicates that the twenty-seven *Fusarium* strains used in this study belong to five genetic clades (**Figure 2** and **Supplementary Figure S1** for a phylogenetic tree including reference strains). These clades harbor members of several *Fusarium* species complexes, namely *F. tricinctum* (clade I - *F. tricinctum, F. acuminatum, F. flocciferum* and *F. torulosum*), *F. oxysporum* (clade II), *F. redolens* (clade III), *F. culmorum* (clade IV) and *F. incarnatum-equiseti* (clade V) (**Figure 2A**).

Volatile organic compounds profiles were generated by SPME-GC/MS for all 27 *Fusarium* strains on malt extract and for seven selected strains grown in soil (**Table 1**). Chromatograms were processed with the Tagfinder software (Luedemann et al., 2008) for alignment. Multivariate analysis was applied to normalized VOC profiles after removal of the signal emitted by the respective control samples (either pure malt extract or non-inoculated soil). A PCoA plot showing all replicates (*n* ≥ 3) per strains illustrates that the VOC profiles of the 27 *Fusarium* strains on malt extract varied by large in accordance to genetic identity (**Figure 2B**). Those strains formed three major clusters. The first cluster included several strains from the *F. tricinctum* species complex, the second cluster all strains of the *F. oxysporum* and *F. redolens* complex and the third complex all remaining strains, including *F. torulosum* and *F. flocciferum* from the *F. tricinctum* complex. Given the axes displayed, 37.4% of total variation could be explained by the model displayed in **Figure 2B**. The same conclusion, that volatilomes depend on genetic identities, can be reached for strains grown in soil (**Figure 2C**). Strains from the *F. tricinctum* species complex were separated from *F. oxysporum* and *F. redolens* strains. Explained variation of the model was 50.9% in **Figure 2C**. Overall this confirms that, regardless of the medium used for fungal growth, species identity has the biggest impact on the VOC profiles of *Fusarium* strains.

### *Fusarium* Strains Increase Primary Root Length in *Arabidopsis* Seedlings

Since genetically distinct *Fusarium* strains differed in their VOC profiles, we hypothesized that these differences might also affect plant growth in specific ways (i.e., stimulation in some cases and inhibition in others). Hence, the influence of selected soil-grown *Fusarium* strains on plant development was tested in a system which prevents physical contact between the test fungus and plant but allows indirect interactions through VOCs (**Figure 1**). The effect of seven *Fusarium* strains and one unclassified Helotiales strain on *Arabidopsis* development (primary root length, root branching and leaf size) was recorded and compared to control seedlings grown in the presence of soil only (no fungus).

**FIGURE 3:**
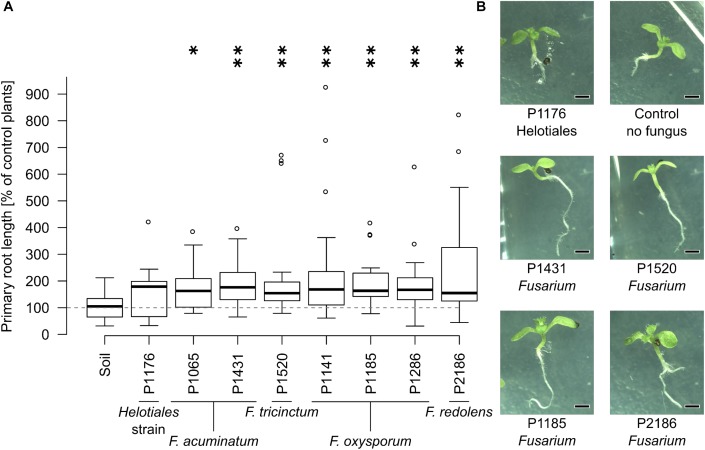
The presence of *Fusarium* increases *Arabidopsis* primary root length. Bioassays were conducted as described in **Figure 1**. **(A)** All soil-grown *Fusarium* strains but not the Helotiales one significantly increased primary root length compared to control seedlings (exposed to soil VOCs only) (Kruskal-Wallis test, ^∗^indicates a *p*-value of ≤0.05, ^∗∗^≤ 0.01, *n* = 14 to 20 seedlings pooled from 3 to 5 Petri dishes). **(B)** Representative 11 days old seedlings grown with or without fungi (scale bar = 1 mm).

Due to high variability between time independent replicates, primary root length and leaf surface area were normalized to control plants (not exposed to fungi). Similarly, root branching was normalized to primary root length. Most fungal strains strongly influenced primary root growth but no significant differences were detected in terms of leaf surface area or root branching (**Supplementary Figure S2**). Specifically, seedlings grown in the presence of all *Fusarium* strains had significantly longer roots than control plants, while the Helotiales strain did not affect primary root development (**Figure 3**). Overall this indicates that all *Fusarium* strains tested had the ability to increase primary root growth. In contrast, the unidentified Helotiales strain did not.

### *Fusarium* spp. Decrease the Emission of Soil VOCs

**FIGURE 4:**
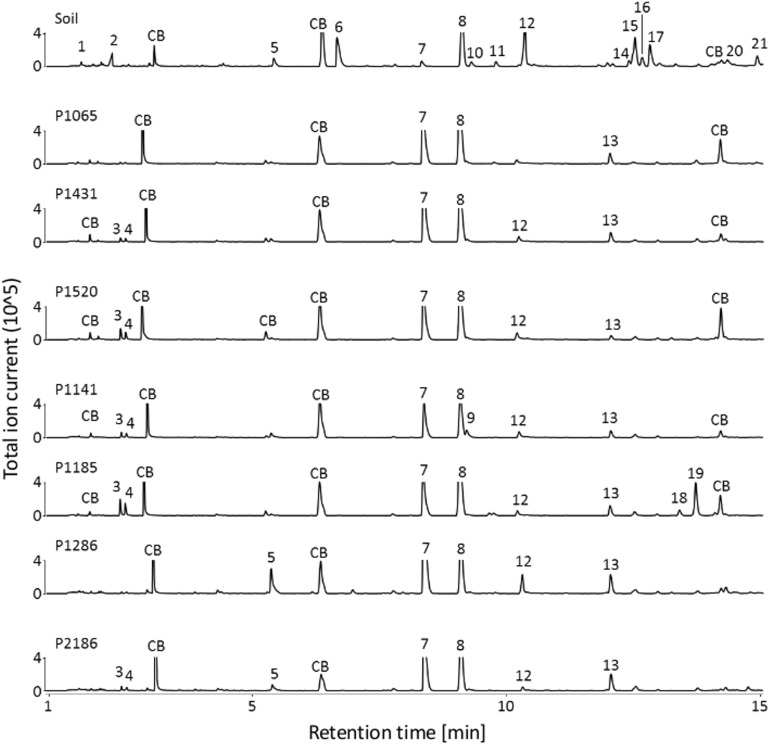
VOCs detected in non-inoculated and *Fusarium*-inoculated soil samples. GC/MS chromatograms (TIC) illustrating the volatile profiles of non-inoculated soil samples and of *Fusarium* inoculated soil samples. *Fusarium* strains overall decreased the number of VOCs detected in the chromatograms compared to non-inoculated soil. CB: column bleed (siloxane derivatives), 1: unidentified, 2: unidentified, 3: 3-methylbutanal, 4: 2-methylbutanal, 5: hexanal, 6: 3-furancarbaldehyde, 7: 4-heptanone^∗^, 8: styrene, 9: 4-heptanol, 10: 2-heptanone, 11: heptanal, 12: methyl-(Z)-N-hydroxybenzenecarboximidate, 13: 7-ethyl-4-nonanone, 14: 2-ethylhexanal, 15: benzaldehyde^∗^, 16: unidentified, 17: 5-methyl-2-furancarboxaldehyde^∗^, 18: unidentified, 19: 1-octen-3-ol, 20: unidentified, 21: octanal^∗^. ^∗^ Fully identified compounds (based on authentic standard, Kovats-retention indices, mass fragmentation), all other compounds were tentatively identified based on Kovats-retention indices and mass fragmentation).

**FIGURE 5:**
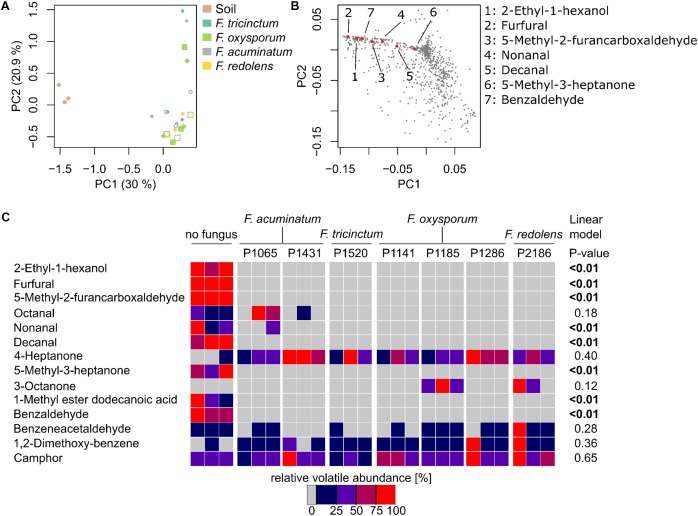
*Fusarium* spp. reduces the emission of soil VOCs. **(A)** Principle Coordinate Analysis ordination (PCoA) based on VOCs emitted by non-inoculated soil and *Fusarium*-inoculated soil samples highlights major differences among non-inoculated and *Fusarium*-inoculated soil (*n* = 3 replicates per strain). **(B)** PCoA illustrating the distribution of TAGs (VOCs) across samples, showing those responsible of the differences observed in (a). Red, numbered dots correspond to identified VOCs. **(C)** Heatmap illustrating the concentration of 14 identified VOCs in *n* = 3 replicates (**Supplementary Figure S5** includes unidentified VOCs as well) and highlighting that non-inoculated soil tends to have a higher concentration of VOCs compared to *Fusarium*-inoculated soil. *P*-values reported on the right side of the heatmap correspond to significant negative correlations among VOC concentration and primary root length (reported in **Figure 3**) obtained through a linear model.

Given the primary root growth stimulation observed with all *Fusarium* strains, we aimed at indentifying the VOC(s) responsible for this effect. VOC profiles of soil-grown *Fusarium* were compared to the one of non-inoculated soil (samples were taken at the end of the biossays documented in **Figure 3**). Chromatograms for soil-grown strains and non-inoculated soil are visible in **Figure 4** (GC/MS data files are available as **Supplementary Data Sheet S3**). Specifically, VOC(s) (TAGs) whose concentration significantly differed among strains and non-inoculated soil were identified by a Kruskal-Wallis test (*p* < 0.05). The resulting data matrix was used to generate a PCoA ordination showing that the VOC profile of non-inoculated soil drastically differed from that of soil inoculated with *Fusarium* (**Figure 5A**). The model overall explained 51% of the data varability. The PCoA scores of individual TAGs (**Figure 5B**) highlight that specific VOCs were responsible for the differences between non-inoculated soil and *Fusarium* inoculated soil seen in **Figure 5A**. TAGs representing VOCs identified by authentic standards are indicatied as red numbered dots in **Figure 5B**.

A heatmap was constructed to vizualize how the concentration of 14 VOCs that could be identified differed among treatments (**Figure 5C**). The corresponding heatmap with an extra 33 unidentified compounds in shown in **Supplementary Figure S5** and corresponding mass spectras can be obtained from GC/MS data files available as **Supplementary Data Sheet S3**. It shows that the concentrations of these VOCs significantly differed between *Fusarium*-inoculated and non-inoculated soil, in most cases due to a reduction of the concentration of numerous VOCs in respect to non-inoculated soil. By contrast, only 3-octanone was absent from non-inoculated soil but present only in two *Fusarium* treatments (**Figure 5C**). Overall this indicates that *Fusarium* spp. have the ability to drastically reduce the concentration of soil VOCs.

To pinpoint VOCs that might be responsible for the root length stimulation described herewith, a linear model was used to test for significant correlations among the concentrations of specific VOCs and primary root length. Seven VOCs, with maximal concentrations in non-inoculated soil, were significantly and negatively correlated to primary root length (**Figure 5C**) and might thus explain root length stimulation observed in the presence of *Fusarium*.

### Mixtures of Synthetic VOCs Decrease Primary Root Length at Concentrations Comparable to Those Emitted by Soil

The linear model of **Figure 5C** suggests that, among identified VOC, seven might be specifically responsible for the primary root length stimulation observed with *Fusarium* (**Figure 3**). The concentrations of these VOCs were quantified from soil by standard addition and ranged at the end of the bioassays from 26 ng g^-1^ to 16 μg g^-1^ soil (wet weight) (**Table 2**).

**FIGURE 6:**
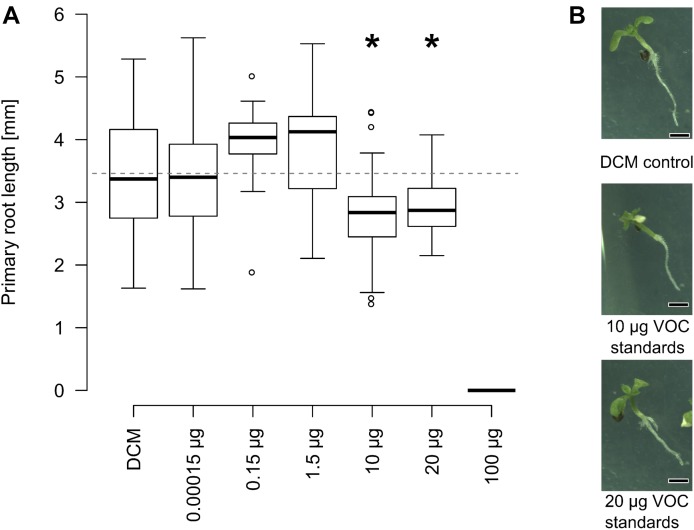
Effect of VOC standards identified in soil on root growth. **(A)** *Arabidopsis* plants were grown on tripartite plates, as described in **Figure 1**. Instead of soil, the second compartment harbored cotton with mixtures of 2-ethyl-1-hexanol, furfural, 5-methyl-2-furancarboxaldehyde, nonanal, decanal, 5-methyl-3-heptanone and benzaldehyde in dichloromethane. Primary root length was mostly unaffected by low concentration, significantly smaller between 10 and 20 μg than plants exposed to dichloromethane without additional VOCs. Germination was fully suppressed at 100 μg (^∗^*p* ≤ 0.05, Kruskal Wallis, *n =* 15–45 seedlings pooled from 6 to 15 Petri dishes). **(B)** Speciemen of 11-days old *Arabidopsis* plants exposed to different volatile blends at the end of the experiment (scale bar = 1 mm).

Bioassays with *Arabidopsis* were subsequently performed using mixtures of the former seven VOCs dissolved in dichloromethane at varying concentrations. Since all those VOCs were emitted by sterilized and non-inoculated soil, a piece of cotton was used in compartment 2 (**Figure 1**) of the bioassay instead of soil. This mixture of VOCs significantly inhibited growth of primary roots at quantities corresponding to 10–20 μg and fully blocked germination from 100 μg onward (**Figure 6**). Overall this demonstrates that a mixture of synthetic soil VOCs effectively decreased primary root length at concentrations comparable to those emitted by soil.

## Discussion

Here we investigated the variability in the VOC profiles of various *Fusarium* fungal species and demonstrated that microbial genetic background and media composition influenced volatile profiles. VOC fingerprints were generated on malt extract for all isolates and on soil for representative members of dominant *Fusarium* clades (**Figure 2**). Medium composition and species identity impacted *Fusarium* VOC emission (**Supplementary Figure S3**), which is in line with previous results illustrating that biotic and abiotic factors can greatly impact secondary metabolism (Blom et al., 2011; Brakhage and Schroeckh, 2011; Schenkel et al., 2015; Schmidt et al., 2016; Lazazzara et al., 2017). Specifically, emission of fungal VOCs might for instance depend on the age/growth stage of mycelium (Naznin et al., 2013; Lee et al., 2015). The influence of growth medium on secondary metabolism can be attributed to the direct availability of precursors in the growth medium or the modulating effects of specific metabolites on secondary metabolism (Zain et al., 2011). Whereas malt extract is a sugar-rich medium (Balcerek et al., 2016), soil is nutrient poor and rich in amino acids and lipids (Pétriacq et al., 2017). These dissimilarities might explain the drastic differences in VOC profiles observed here for the same species grown on soil or malt extract (**Figures 2B, 5A, Supplementary Figures S3, S4**). Furthermore, the fact that soil contains a large pool of uncharacterized metabolites (Pétriacq et al., 2017) might explain why the structure of numerous VOCs could not be elucidated based on existing mass spectral databases. VOCs emitted by soil indeed depend on a plethora of variables including environmental and soil physico-chemical factors (Insam and Seewald, 2010). Elucidating the structure of novel soil VOCs and understanding how they are formed will be a challenge for future studies.

Bioassays investigating VOC-based interactions between soil-grown *Fusarium* and *Arabidopsis* highlighted the ability of the *Fusarium* genus to increase primary root length. This growth promoting effect was not observed for the unidentified Helotiales strain, suggesting a high impact of genetic background. It has been hypothesized that fungi with different lifestyles (i.e., pathogens, saprophytes, symbiotic) might emit different volatiles (Müller et al., 2013; Schenkel et al., 2015), which could in turn partially explain plant phenotype. The dataset presented here is however not optimal to validate this hypothesis, first because the Helotiales strain used here has not been fully characterized and, second because numerous *Fusarium* isolates can switch their lifestyle depending on plant hosts and environmental conditions (Kia et al., 2017; Lofgren et al., 2018). Thus, attributing a strict pathogenic/saprotrophic/endophytic lifestyle to these strains would be difficult and likely misleading.

Volatile organic compound-based root length stimulation comparable to the one reported here (i.e., 49–126% increase compared to control plants) have been described by others (Bitas et al., 2015; Ditengou et al., 2015). By contrast to primary root length, leaf area and root branching of the 11-days-old seedlings were not significantly affected by the presence of *Fusarium* strains in this study. This is in opposition with earlier results that reported increased leaf size and root branching in *Arabidopsis* seedlings of comparable age along with primary root length stimulation (Ryu et al., 2003; Splivallo et al., 2009; Bitas et al., 2015; Ditengou et al., 2015). This might indicate that under our experimental conditions, *Fusarium* specifically stimulated primary root length or that root branching and leaf surface would only have been affected at a later plant developmental stage. The root length shortening observed with *Arabidopsis* could be attributed to seven VOCs that showed bioactivity from 70 μg (a mixture of 10 μg each) up. This is comparable to quantities of bioactive VOCs reported by others (Ryu et al., 2003; Zou et al., 2010; Blom et al., 2011; Park et al., 2015). Additionally, one of the bioactive VOC tested here (2-ethyl-1-hexanol) is phytotoxic to ornamental plants (Hörmann et al., 2018). Demonstrating whether similar effects occur under field conditions will be a challenge that will require to carefully take soil parameters as well as source and sink mechanisms under consideration.

Lastly, our results indicate that *Fusarium* has the capacity to decrease soil VOCs. The mechanism behind this phenomenon is elusive, yet the ability of microbes to consume VOCs has been occasionally described. For instance, microbial communities are able to degrade VOCs in soil including benzene and toluene under aerobic conditions (Yoshikawa et al., 2017). Tracer experiments with radioactive carbon revealed that the labelled monoterpene geraniol was metabolized by unidentified soil/rhisophere microbes and transformed into another monoterpene (limonene) (Owen et al., 2007). Mono- and sesquiterpenes can also serve as carbon source to rhizobacteria (Best et al., 1987; Kleinheinz et al., 1999; Del Giudice et al., 2008; Schulz-Bohm et al., 2015, 2017). It has also been suggested that VOCs might act as carbon source for fungal growth on carbon-poor substrate (Cale et al., 2016). Besides being metabolized or transformed, soil VOCs might also be adsorbed to fungal hyphae. Demonstrating the exact mechanism that leads to the decrease of specific VOCs by *Fusarium* will require combining labeling experiments to metabolomics in order to understand the fate of specific VOCs.

Overall our results constitute an important paradigm shift in plant-microbial interactions since they suggest that microbes shall not only be considered as the possible emitter of bioactive VOCs, but also as a possible sink. Shedding light on the implications of VOC degradation by microbes will require understanding their breakdown mechanisms which remains elusive. Nevertheless, this finding might have important implications in plant-microbial interactions and suggest that considering the soil volatilome might help to predict plant productivity.

## Author Contributions

RS and DS designed all the experiments. DS performed all the experiments with input from AB for the bioassays on soil. JM-V provided plant and fungal material and contributed to fungal characterization. DS, RS, and JM-V analyzed the data and wrote the manuscript.

## Conflict of Interest Statement

The authors declare that the research was conducted in the absence of any commercial or financial relationships that could be construed as a potential conflict of interest.

## Supplementary Material

FIGURE S1 Phylogenetic tree of *Fusarium* strains used in this study and reference strains. Phylogenetic tree of 27 *Fusarium* strains (**Table 1**) and reference strains [FUSARIUM-ID database (Geiser et al., 2004)] based on *tef-1*α sequences. Numbers at nodes indicates bootstrap values (1,000 replications).

FIGURE S2 The presence of *Fusarium* does not affect *Arabidopsis* root branching or leaf surface area. Bioassays were conducted as described in **Figure 1**. No significant differences in leaf surface area or root branching (number of secondary roots) were observed between *Arabidopsis* seedlings exposed to VOCs from non-inoculated soil or *Fusarium* or Helotiales-inoculated soil (statistics: Kruskal-Wallis test, *p* ≤ 0.05).

FIGURE S3 Comparison the effect of substrate on the VOC profile of *Fusarium*. Cluster analysis of the VOC profile of seven *Fusarium* strains grown either on malt extract (3–4 replicates per strain) or soil (3 replicates per strain). A representative VOC profile for each strain was generated by considering only TAGs (VOCs) which consistently appeared in all replicates, and these representative profiles were used to compute the dendrogram presented here. Signal present in the respective controls (i.e., soil or pure malt extract) was removed. TAGs transformed into binary data (i.e., presence–absence) were used to generate a cluster tree using Jaccard similarity index. ∙ represent nodes with ≥70% (1,000 bootstraps).

FIGURE S4 VOC profiles of *Fusarium* strain P1065 on malt extract and soil. The two upper chromatograms represent the VOC profile of strain P1065 on malt extract and the profile of the control (malt extract without fungus), whereas the two lower chromatograms illustrate the VOC emitted by the same fungus on soil (or the soil control). These results exemplify the influence of the medium (malt extract versus soil) on microbial VOC emission. VOCs were identified or tentatively identified as: (1) 1-hexanol; (2) 4-heptanone; (3) styrene; (4) 7-ethyl-4-noanone; (5) benzaldehyde; (6) 1,3,5-trimethylbenzene; (7) 5-methyl-2-furancarboxaldehyde; (8) 1-heptanol.

FIGURE S5 VOC profiles of soil samples at the end of the bioassay. Heatmap illustrating the concentration of 14 identified VOCs and 33 not identified VOCs in *n* = 3 replicates per fungal strain and highlighting that non-inoculated soil tends to have a higher concentration of VOCs compared to *Fusarium*-inoculated soil.

DATA SHEET S3 GC/MS data for non-inoculated and *Fusarium* inoculated soil.
