# Supplementary material for: Fungi Indirectly Affect Plant Root Architecture by Modulating Soil Volatile Organic Compounds
Source: Front Microbiol. 2018 Aug 13;9:1847. doi: 10.3389/fmicb.2018.01847 (PMC6099090; doi:10.3389/fmicb.2018.01847)
Supplement: FIGURE S1 — Phylogenetic tree of Fusarium strains used in this study and reference strains. Phylogenetic tree of 27 Fusarium strains (Table 1) and reference strains [FUSARIUM-ID database (Geiser et al., 2004)] based on tef-1α sequences. Numbers at nodes indicates bootstrap values (1,000 replications). [file Data_Sheet_1.pdf]

Fungi indirectly affect plant root architecture by modulating soil volatile organic compounds

Schenkel Denis[1,3], Maciá-Vicente Jose G.[2,3], Bissell Alexander[1], Splivallo Richard[1,3]*

1 Institute for Molecular Biosciences, Goethe University Frankfurt, Max-von-Laue Str. 9, 60438 Frankfurt am Main, Germany

2 Institute of Ecology, Evolution and Diversity, Goethe University Frankfurt, Max-von-Laue Str. 13, 60438 Frankfurt am Main, Germany

3 Integrative Fungal Research Cluster, Frankfurt, Georg-Voigt-Str. 14-16, 60325 Frankfurt, Germany

*corresponding author: richard.splivallo@a3.epfl.ch

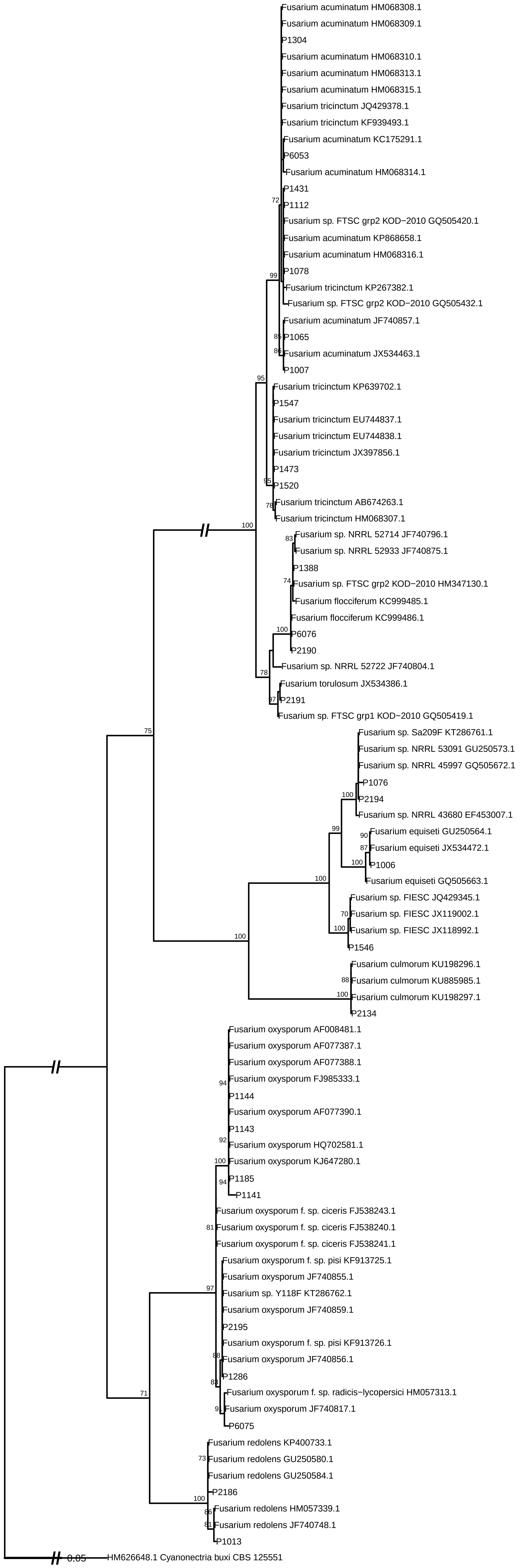

Figure S1. Phylogenetic tree of Fusarium strains used in this study and reference strains.

Phylogenetic tree of 27 Fusarium strains (Table 1) and reference strains (FUSARIUM-ID database (Geiser et al., 2004)) based on partial tef-1α sequences. Numbers at nodes indicates bootstrap values (1000 replications)
