# Supplementary material for: Fungi Indirectly Affect Plant Root Architecture by Modulating Soil Volatile Organic Compounds
Source: Front Microbiol. 2018 Aug 13;9:1847. doi: 10.3389/fmicb.2018.01847 (PMC6099090; doi:10.3389/fmicb.2018.01847)
Supplement: FIGURE S2 — The presence of Fusarium does not affect Arabidopsis root branching or leaf surface area. Bioassays were conducted as described in Figure 1. No significant differences in leaf surface area or root branching (number of secondary roots) were observed between Arabidopsis seedlings exposed to VOCs from non-inoculated soil or Fusarium or Helotiales-inoculated soil (statistics: Kruskal-Wallis test, p ≤ 0.05). [file Data_Sheet_2.pdf]

Fungi indirectly affect plant root architecture by modulating soil volatile organic compounds

Schenkel Denis[1,3], Maciá-Vicente Jose G.[2,3], Bissell Alexander[1], Splivallo Richard[1,3*]

[1] Institute for Molecular Biosciences, Goethe University Frankfurt, Max-von-Laue Str. 9, 60438 Frankfurt am Main, Germany
[2] Institute of Ecology, Evolution and Diversity, Goethe University Frankfurt, Max-von-Laue Str. 13, 60438 Frankfurt am Main, Germany
[3] Integrative Fungal Research Cluster, Frankfurt, Georg-Voigt-Str. 14-16, 60325 Frankfurt, Germany

*corresponding author: richard.splivallo@a3.epfl.ch

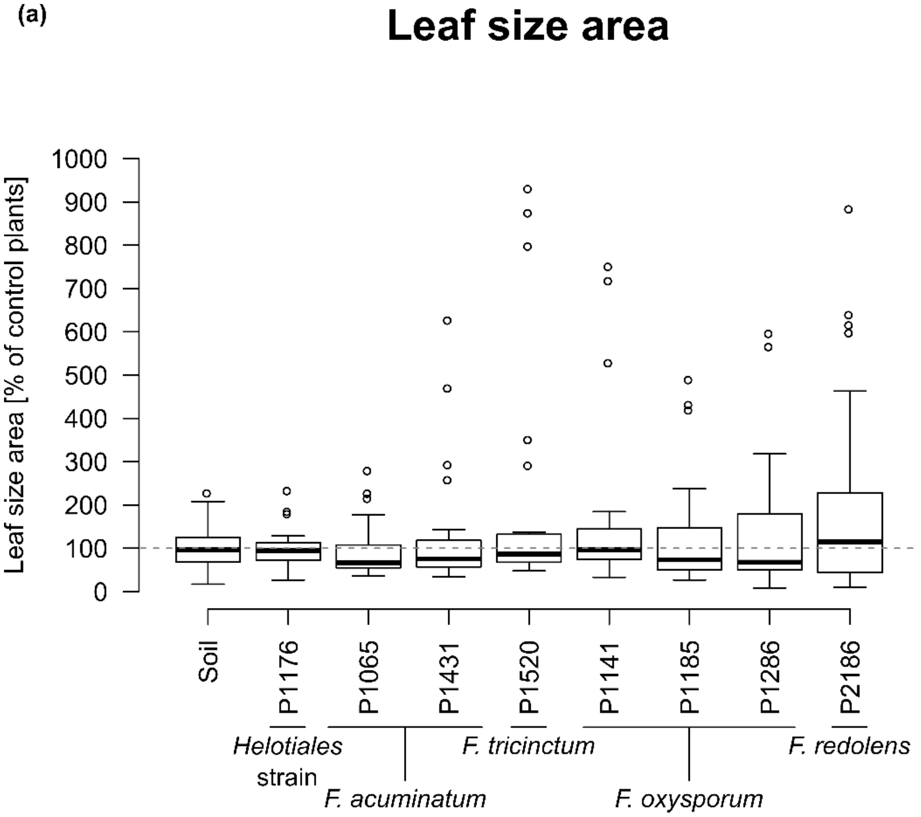

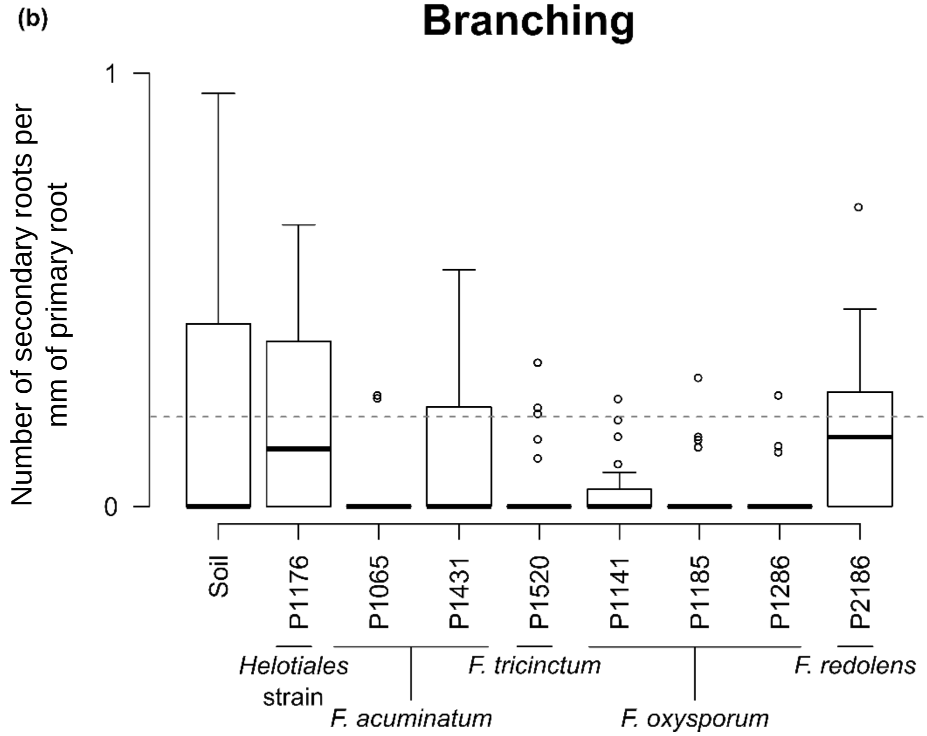

Figure S2. The presence of *Fusarium* does not affect *Arabidopsis* root branching or leaf surface area.
Bioassays were conducted as described in Fig. 1. No significant differences in leaf surface area or root branching (number of secondary roots) were observed between *Arabidopsis* seedlings exposed to VOCs from non-inoculated soil or *Fusarium* or Helotiales-inoculated soil (statistics: Kruskal-Wallis test, $p \leq 0.05$)

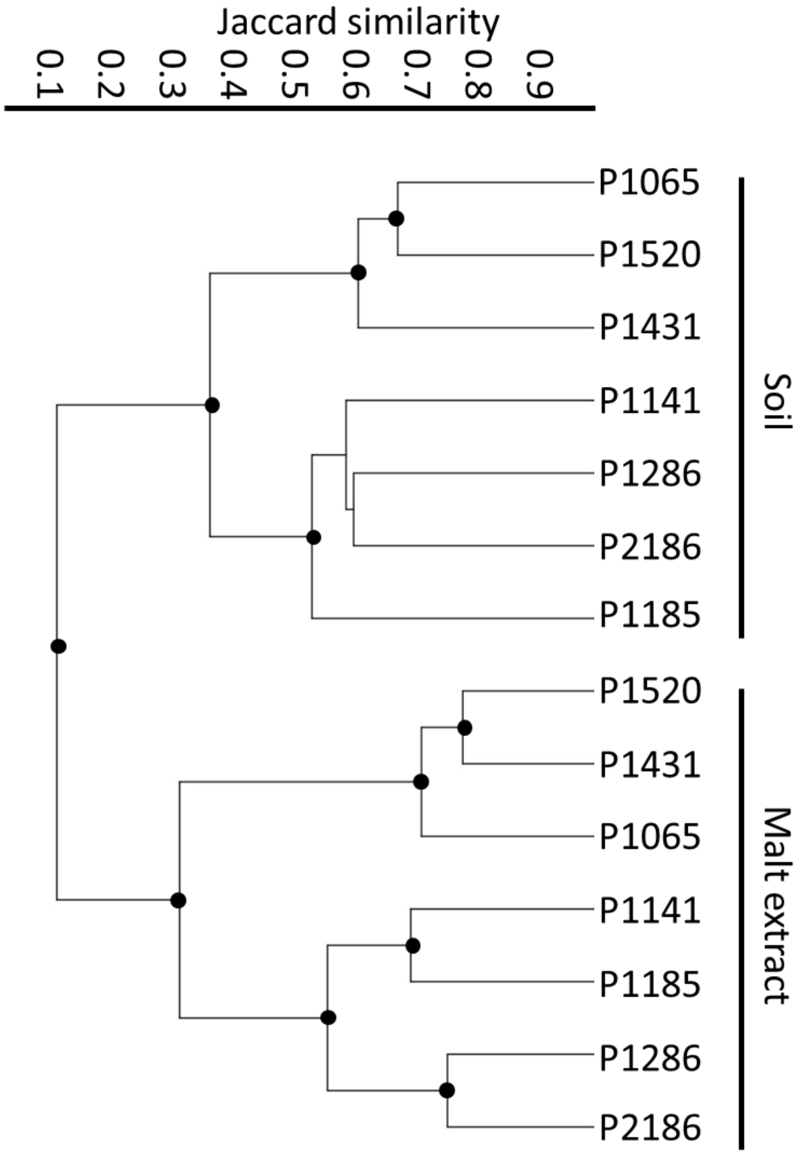

Figure S3. Comparison the effect of substrate on the VOC profile of *Fusarium*.

Cluster analysis of the VOC profile of seven *Fusarium* strains grown either on malt extract (3-4 replicates per strain) or soil (3 replicates per strain). A representative VOC profile for each strain was generated by considering only TAGs (VOCs) which consistently appeared in all replicates, and these representative profiles were used to compute the dendrogram presented here. Signal present in the respective controls (i.e. soil or pure malt extract) was removed. TAGs transformed into binary data (i.e. presence-absence) were used to generate a cluster tree using Jaccard similarity index. ● represent nodes with ≥70% (1,000 bootstraps).

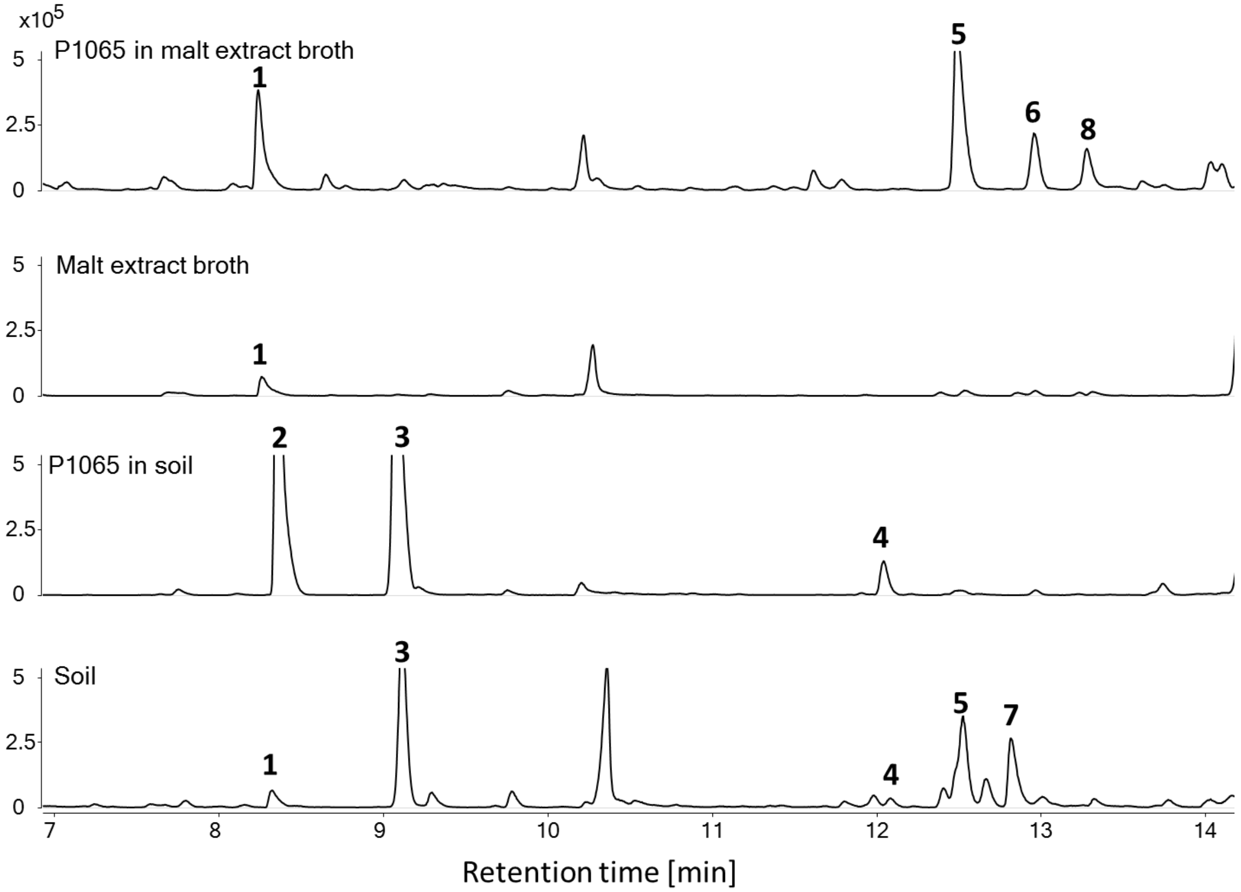

Figure S4. VOC profiles of *Fusarium* strain P1065 on malt extract and soil.

The two upper chromatograms represent the VOC profile of strain P1065 on malt extract and the profile of the control (malt extract without fungus), whereas the two lower chromatograms illustrate the VOC emitted by the same fungus on soil (or the soil control). These results exemplify the influence of the medium (malt extract versus soil) on microbial VOC emission. VOCs were identified or tentatively identified as: (1) 1-hexanol; (2) 4-heptanone; (3) styrene; (4) 7-ethyl-4-noanone; (5) benzaldehyde, (6) 1,3,5-trimethylbenzene; (7) 5-methyl-2-furancarboxaldehyde; (8) 1-heptanol.

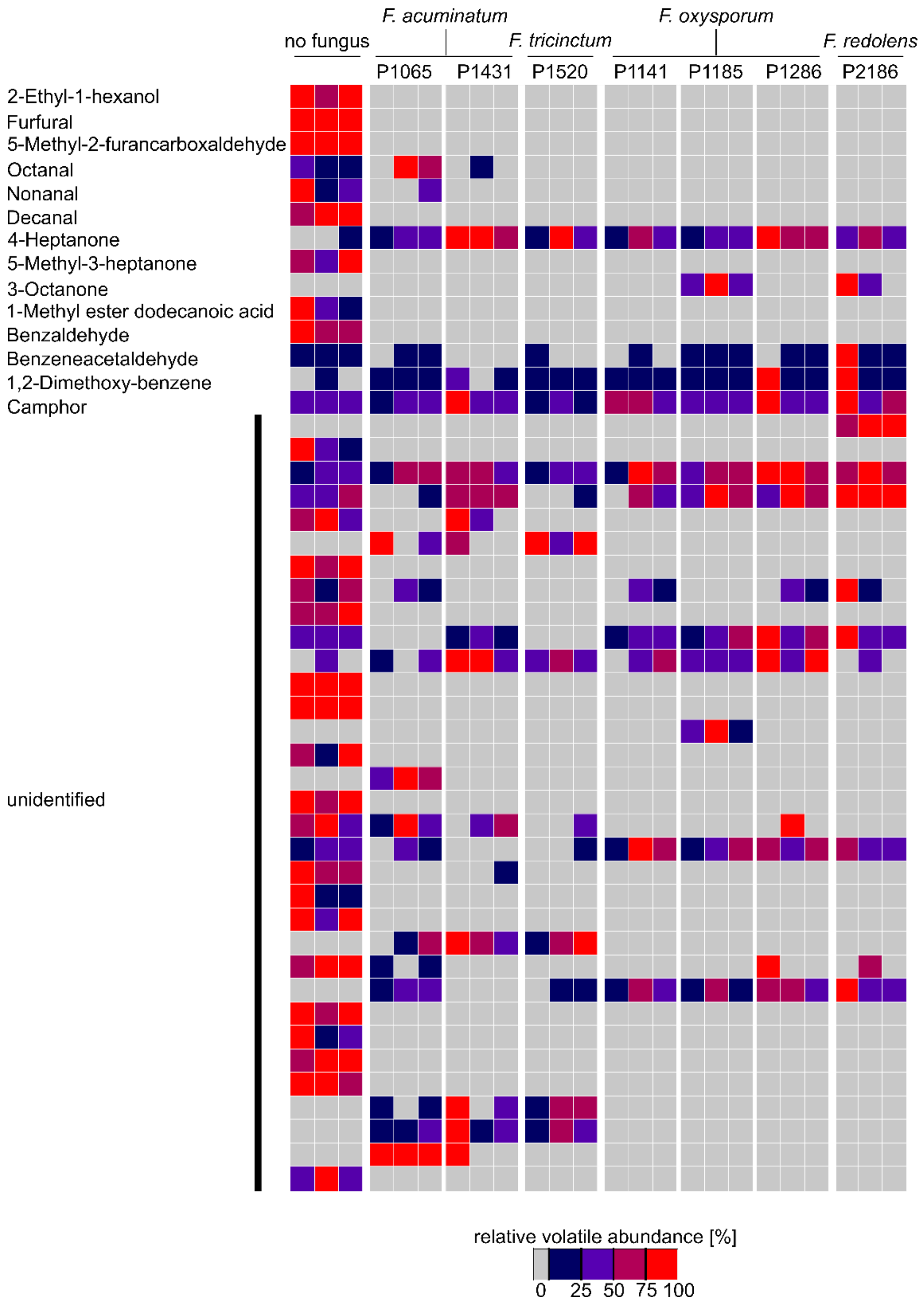

Figure S5. VOC profiles of soil samples at the end of the bioassay
Heatmap illustrating the concentration of 14 identified VOCs and 33 not identified VOCs in n = 3 replicates per fungal strain and highlighting that non-inoculated soil tends to have a higher concentration of VOCs compared to *Fusarium*-inoculated soil.
